# Long-term Treatment with Low-Dose Caffeine Worsens BPSD-Like Profile in 3xTg-AD Mice Model of Alzheimer’s Disease and Affects Mice with Normal Aging

**DOI:** 10.3389/fphar.2018.00079

**Published:** 2018-02-15

**Authors:** Raquel Baeta-Corral, Björn Johansson, Lydia Giménez-Llort

**Affiliations:** ^1^Institut de Neurociències, Universitat Autònoma de Barcelona, Barcelona, Spain; ^2^Department of Psychiatry and Forensic Medicine, Universitat Autònoma de Barcelona, Barcelona, Spain; ^3^Department of Molecular Medicine and Surgery, Karolinska Institutet, Solna, Sweden; ^4^Department of Geriatrics, Karolinska University Hospital, Solna, Sweden

**Keywords:** aging, anxiety, memory, NPS, BPSD, circadian activity, translational, long-term effects

## Abstract

Coffee or caffeine has recently been suggested as prophylaxis for dementia. Although memory problems are hallmarks of Alzheimer’s disease, this dementia is also characterized by neuropsychiatric symptoms called Behavioral and Psychological Symptoms of Dementia (BPSD). The impact of preventive/therapeutic strategies on both cognitive and non-cognitive symptoms can be addressed in the 3xTg-AD mice, since they exhibit cognitive but also BPSD-like profiles. Here, we studied the long-term effects of a low dose of caffeine in male 3xTg-AD mice and as compared to age-matched non-transgenic (NTg) counterparts with normal aging. Animals were treated (water or caffeine in drinking water) from adulthood (6 months of age) until middle-aged (13 months of age), that in 3xTg-AD mice correspond to onset of cognitive impairment and advanced stages, respectively. The low caffeine dosing used (0.3 mg/ml) was previously found to give a plasma concentration profile in mice roughly equivalent to that of a human coffee drinker. There were significant effects of caffeine on most behavioral variables, especially those related to neophobia and other anxiety-like behaviors, emotionality, and cognitive flexibility. The 3xTg-AD and NTg mice were differently influenced by caffeine. Overall, the increase of neophobia and other anxiety-related behaviors resulted in an exacerbation of BPSD-like profile in 3xTg-AD mice. Learning and memory, strongly influenced by anxiety in 3xTg-AD mice, got little benefit from caffeine, only shown after a detailed analysis of navigation strategies. The worsened pattern in NTg mice and the use of search strategies in 3xTg-AD mice make both groups more similar. Circadian motor activity showed genotype differences, which were found to be enhanced by caffeine. Selective effects of caffeine on NTg were found in the modulation of behaviors related to emotional profile and risk assessment. Caffeine normalized splenomegaly of 3xTg-AD mice, a physical indicator of their impaired peripheral immune system, and trended to increase their corticosterone levels. Our observations of adverse caffeine effects in an Alzheimer’s disease model together with previous clinical observations suggest that an exacerbation of BPSD-like symptoms may partly interfere with the beneficial cognitive effects of caffeine. These results are relevant when coffee-derived new potential treatments for dementia are to be devised and tested.

## Introduction

Caffeine, a non-selective A1 and A2A receptor antagonist, is one of the most consumed drugs all over the world. The average consumption of caffeine in humans is around 300–400 mg/day (three to four cups of coffee) and its effects in several physiological functions, such as locomotion, sleep, and cardiovascular function, depend on the dose and duration of the consumption (Fredholm et al., 1999, 2017; Fredholm, 2007). A large part of the cognitive enhancing properties of caffeine is due to its indirect action on arousal, mood, and concentration (reviewed by Nehlig, 2010). Thus, low doses of caffeine (20–200 mg/day) have been associated with positive effects on subjective mood: wellbeing, confidence, motivation, alert, security, efficiency, concentration, and desire for socialization (see Griffiths et al., 1990; Silverman et al., 1994). In this low range, caffeine (up to 300–400 mg) has also a stimulating action with biphasic motor effects (Fredholm et al., 1999). However, restraint from moderate or high intake of coffee (more than four cups a day) is recommended due to negative effects of caffeine on pregnancy, risk of osteoporosis, cardiovascular problems, anxiety, sleep disturbances, and alterations in physiological functions such as locomotion (Fredholm et al., 1999; Johansson et al., 2001; Giménez-Llort et al., 2005; Fredholm, 2007; Hermansen et al., 2012).

In the last decade, a neuroprotective role of caffeine and other compounds of coffee such as theophylline has been postulated and it is of a growing interest (Maia and de Mendonça, 2002; Chen et al., 2010; Eskelinen and Kivipelto, 2010; Cao et al., 2012). For instance, the study “Cardiovascular Risk Factors, Aging and Dementia” (Eskelinen et al., 2009) indicated that consumption of three to five cups of coffee daily average age of the population is associated in 65% of cases, with a lower risk of developing dementia in the future. Although the whole complexity of aging process is still unknown, the use of caffeine to treat cognitive deficits associated with natural aging and those in Alzheimer’s disease is foreseen as promising. With that, a substantial number of studies have been published suggesting preventive effects of coffee or caffeine on Alzheimer’s disease (e.g., Arendash et al., 2006; Solfrizzi et al., 2015; Kolahdouzan and Hamadeh, 2017; Oñatibia-Astibia et al., 2017; Wierzejska, 2017).

The role of caffeine as a possible protective agent is supported by the pharmacological action of caffeine blocking adenosine A2A receptors, which show an aberrant expression and function in aging and related diseases (Marques et al., 2011). At the experimental level, long-term caffeine treatment has been demonstrated to ameliorate cognitive impairment in animal models of Alzheimer disease: βA-injection mouse models (Dall’Igna et al., 2007; Canas et al., 2009) and transgenic mouse models including APP (Arendash et al., 2006; Cao et al., 2009; Chu et al., 2012), APP/PS1 (Cao et al., 2011; Han et al., 2013), and more recently in a tau transgenic model (Laurent et al., 2014). Most importantly, because among the underlying mechanisms the reduction of amyloid beta production is postulated (Arendash et al., 2006). Interestingly, age-like HPA-axis dysfunction has been related to overactivation of caffeine-binding adenosine A2A receptors in rats mimicking the upregulation found in the forebrain of aged and AD patients, and their direct regulatory action on glucocorticoid receptor function (Batalha et al., 2016).

The main clinical manifestation of dementia is a decline in cognitive function. However, neuropsychiatric symptoms (NPS) are quite prevalent among the patients since early stages of Alzheimer’s disease (Reisberg et al., 1987) and show a clear trend toward increasing their frequency with the progress of the disease (Piccininni et al., 2005). The symptoms, also referred as “Behavioral and Psychological Symptoms of Dementia” (BPSD), may include depression, apathy, hallucinations, delusions, agitation, aggression, and sleep disturbances. This wide array of NPS or BPSD is considered a strong source of distress and burden for AD patients and caregivers. The treatment of these NPS is a major challenge (Wang et al., 2016) as it is the understanding of the pathophysiology underlying their comorbidity in Alzheimer’s disease (e.g., reviewed by Corrêa-Velloso et al., 2018). At the experimental level, research in animal models of Alzheimer’s disease has focused on the cognitive deficits while few of them have also considered their non-cognitive profile (reviewed by Giménez-Llort et al., 2007). Since 2006, our laboratory has been devoted to characterize the cognitive but also the non-cognitive symptoms (i.e., anxiety, phobias, bizarre behaviors, hyperactivity, disinhibition, apathy and motivation, persistence of behaviors, and diurnal rhythm disturbances) in the homozygous 3xTg-AD mice created by LaFerla (Oddo et al., 2003). As we have consistently reported (e.g., Giménez-Llort et al., 2006, 2008, 2010; Baeta-Corral and Giménez-Llort, 2014, 2015; Torres-Lista and Giménez-Llort, 2014, 2015; Manuel et al., 2016), these animals show a noticeable BPSD-like profile. Recently, depressive-like profile has also been reported in the 3xTg-AD mice (Romano et al., 2015), early symptoms bearing some resemblance to bipolar disorder have also been noticed (Corrêa-Velloso et al., 2018), and the effects of preventive/therapeutical strategies on such BPSD-like symptoms have began to be studied (García-Mesa et al., 2011, 2012; Blázquez et al., 2014; Cañete et al., 2015; Torres-Lista and Giménez-Llort, 2015; Sabogal-Guáqueta et al., 2017).

In our focus of interest, the 3xTg-AD mice and their non-transgenic (NTg) counterparts with normal aging may be useful to investigate whether the aging process or the presence of an anxiety-like BPSD profile may modify the output of the potential therapeutic benefits of caffeine. The effects of caffeine on sensorimotor performance (open field, balance beam, string agility) and anxiety level [elevated plus-maze (EPM)] have been addressed by Arendash and Cao (2010), in the APP_Swe_ mice. In the present work, we explored the effects of a long-term (7 months) chronic treatment with a very low oral dose of caffeine (0.3 mg/kg) starting at the adulthood until the middle age (from 6 to 13 months of age) of 3xTg-AD mice, and as compared to age-matched NTg mice. In the transgenic mice, these ages correspond to the onset and advanced stages of the disease, respectively (Oddo et al., 2003). Since adenosine receptors are involved in neuronal but also non-neuronal mechanisms, including immunoendocrine responses, the effects of chronic treatment were assessed on sensorimotor functions, physiology [body weight (BW), circadian motor activity, and survival], immunoendocrine system (spleen size and corticosterone), and behavior (exploratory activity, bizarre movements, emotional and anxiety-like behaviors, risk assessment, visual perceptual learning, and reference spatial learning and memory). The effects of caffeine on other BPSD such as apathy/depression were indirectly monitored by means of opposed behaviors [exploration in the activity tests (ACT), floating in the Morris water maze (MWM)] and tests [hole-board (HB) for novelty seeking, cue learning with a visual platform in the water maze].

## Materials and Methods

### Animals

Homozygous triple-transgenic 3xTg-AD mice harboring PS1/_M146V_, APP_Swe_, and tau_P301L_ transgenes were genetically engineered at the University of California, Irvine, as previously described (Oddo et al., 2003). Briefly, two independent transgenes (encoding human APP_Swe_ and human tau_P301L_, both under control of the mouse Thy1.2 regulatory element) were co-injected into single-cell embryos harvested from homozygous mutant PS1M146V knock-in (PS1KI) mice. The PS1 knock-in mice were originally generated as a hybrid C57BL/6 x 129.

Thirty-eight 6-month-old 3xTg-AD mice and C57BL/6 x 129 mice from 15 l of a breeding program that was established in our laboratory at the Medical Psychology Unit, Universitat Autònoma de Barcelona, were used in this study. All the animals were housed three to four per cage and maintained (Makrolon, 35 × 35 × 25 cm) under standard laboratory conditions (12 h light:dark, cycle starting at 8:00 h, food and water available *ad libitum*, 22 ± 2°C, 50–60% humidity). The circadian activity was recorded during one whole light–dark (LD) period, and the rest of the tests from 9:00 to 13:00 h.

This study was carried out in accordance with the recommendations of Animals in Research: Reporting In Vivo Experiments (ARRIVE) guidelines developed by the NC3Rs (Kilkenny et al., 2010) and the Spanish legislation on “Protection of Animals Used for Experimental and Other Scientific Purposes” and the European Communities Council Directive (2010/63/EU) on this subject. The protocol CEEAH 2481/DMAH 8700 entitled “Risk factors and preventive/therapeutical strategies in Alzheimer’s disease: studies in triple-transgenic 3xTg-AD mice” was approved by Departament de Medi Ambient i Habitatge, Generalitat de Catalunya.

### Caffeine Treatment

Mice were allowed to consume *ad libitum* either drinking water or caffeinated drinking water at 0.3 mg/ml (Sigma, St. Louis, MO, United States) beginning at 6 months of age, considered the age of onset of cognitive symptoms in this animal model. The experimental design consisted in the following groups: NTg vehicle, NTg caffeine, Tg vehicle, and Tg caffeine (*n* = 8–10, in each group). Caffeine treatment was continued throughout behavioral testing until the end of the experiment (13 months of age).

It has been previously confirmed that this treatment regimen leads to a 1.5 mg daily dose in a mouse and it is equivalent to an approximately 500 mg daily caffeine intake (approximately five cups of coffee) by a human (Johansson et al., 1996; Arendash et al., 2006). A plasma concentration of caffeine about 30 μM (*circa* three cups of coffee daily) has been recommended to probe the beneficial effects of caffeine on cognition (Costenla et al., 2010).

### Behavioral Assessments

The effects of the chronic caffeine treatment on physical and behavioral profile of 3xTg-AD mice and their NTg counterparts were assessed at 13 months of age, considered advanced stages of disease in this animal model. The battery of behavioral tests consisted in the evaluation of sensorimotor functions and a series of classical unconditioned tasks measuring locomotion and exploratory activity, anxiety-like behaviors, and cognitive functions.

#### Day 1. Corner Test (CT) and Open-Field (OF) Test

Neophobia was evaluated in the corner test (CT) for 30 s. Animals were individually placed in the center of a clean standard home cage, filled with wood shave bedding. Number of corners visited, latency to realize the first rearing, and the number of rearings were recorded.

Immediately after the CT, mice were placed in the center of an open field (homemade woodwork, white box, 50 × 50 × 20 cm) and observed for 5 min. The temporal profile of the following sequence of behavioral events was recorded: duration of freezing behavior, latency to leave the central square and that of entering the peripheral ring, as well as latency and total duration of self-grooming behavior. Horizontal (crossings of 10 × 10 cm squares) and vertical (rearings with a wall support) locomotor activities were also measured. Bizarre behaviors observed in this test were also measured according to the previous reported criterion (Baeta-Corral and Giménez-Llort, 2014). During the tests, defecation boli and urination were also recorded.

#### Day 2. Hole-Board (HB) Test

Mice were placed in the center of the apparatus (woodwork white box of 32 × 32 × 32 cm) with four holes (3 cm diameter) equally spaced in the floor of the HB. In the exploratory behavior, non-goal-directed (rearings) and goal-directed (head-dips) exploratory activities were measured for 5 min. Moreover, the time spent head-dipping, the latencies of first movement, first dipping, and to explore the four different holes (this last one was established as criterion of the four holes exploration) were also measured. Repetition of already visited holes before reaching the criterion was considered as errors and the total number was measured. Defecation boli were also recorded.

#### Day 3. Dark–Light Box (DLB) Test

The dark–light box (DLB) test (Panlab, S.L., Barcelona, Spain) consists of a two-compartment box (black and dark, 27 × 18 × 27 cm; white and illuminated 20 W, 27 × 27 × 27 cm) connected by an opening (7 × 7 cm). The mice were placed into the dark compartment and observed for 5 min. Latency to enter into the lit compartment (all four paws criterion), number of entries, total time spent, and distance covered as well as number of rearings and groomings in this compartment were noted. Risk assessment was measured by means of the latency and number of stretch attendances toward the lit area. Defecation boli and urination in each of both compartments were measured.

#### Day 4. Elevated Plus-Maze (EPM) Test

The plus-maze (woodwork, black Plexiglass) consisted of two enclosed arms (EAs, 30.3 × 5.3 × 15 cm, transparent walls) and two open arms (OAs, 30.3 × 5 cm) forming a square cross with a 5.3 × 5 cm square center piece. The apparatus was elevated 40 cm above the floor. The animal was placed in the center of the plus-maze facing one of the OAs. The number of entries (all four paws criterion) into OA and EA, the time spent in each arm, and defecation boli were recorded for 5 min. The anxiety index TOA/(TOA+TEA) was calculated as time in the OA/(time in the OA + time in the EA).

#### Day 5. T-Maze (TM) Test

The apparatus consists in a T-shaped maze (two short arms of 25 × 8 cm and a long arm of 30 × 8 cm). The working memory paradigm assessed in the T-maze (TM) consisted in two consecutive trials: one forced choice in the first trial and one free choice (recall trial) in the second trial, with a 90-s intertrial interval. In the forced choice, only one of the arms according to a random order and contrabalanced in each group was accessible. The animal was placed inside the “vertical” arm of the maze with its head facing the end wall and it was allowed to explore the maze. After spending 20 s in the accessible arm, the animal was put back into the home cage starting box. This 20 s period was established as the learning criterion. In the recall trial, the animal was allowed to explore the maze in a free choice trial where both arms were accessible. The arm chosen by the mice and the time spent in each arm during the free choice was recorded. The choice of the already visited arm in the previous trial before exploring the arm that was inaccessible was considered as an error and the total number was calculated. Also the time spent to explore the three arms of the maze was recorded. Finally, defecation boli and urination were also recorded.

#### Day 6. Marble Test (MB)

The procedure for marble test (MB) was adopted with minor modifications from that originally described by Broekkamp et al. (1986). Mice were placed individually in a standard home cage (Macrolon, 35 × 35 × 25 cm). The cage contained six glass marbles (dimensions 1 × 1 × 1 cm) evenly spaced making a triangle (three rows of three, two, and one marbles per row only in the left area of the cage) on a 5-cm thick layer of sawdust. The mice were left in the cage with marbles for a 30-min period after which the test was terminated by removing the mice and counting the number of marbles: intact (the number of marbles untouched), rotated (the number of marbles rotated 90° or 180°), half-buried (the number of marbles at least ½ buried by sawdust), and buried (the number of marbles 100% buried by sawdust).

#### Day 7. Body Weight (BW) and Sensorimotor Functions (SMT)

The physical condition of the mice was evaluated by their BW and sensorimotor functions. Visual reflex and posterior legs extension reflex were measured three times by holding the animal by its tail and slowly lowering it toward a black surface. Motor coordination and equilibrium were assessed twice (20-s trials) in two consecutive rod tasks of increasing difficulty. The distance covered and the latency to fall off a wooden (1.3 cm wide) and a metal wire (1 cm diameter) rod (both, 1 m long) were recorded. The hanger test was used to measure prehensibility and motor coordination by the distance covered and the number of elements of support and the latency to fall. The animal was allowed to cling with its forepaws from the middle of a horizontal wire (2 mm diameter, 40 cm length, divided into eight 5 cm segments) for two trials of 5 s. A third trial of 60 s was used to complement these measures with that of muscle strength or resistance. All the apparatus were suspended 40 cm above a padded table.

#### Days 8–15. Circadian Motor Activity Test (ACT)

Three mice per day were tested for 23 consecutive hours (beginning at 15.00 h, periods of 30 min) in a multicage activity meter system (three cages simultaneously, Actitrack, Panlab, S.L., Barcelona, Spain) set to measure spontaneous locomotor activity. Each testing cage (Macrolon, 35.3 × 35.3 × 25 cm) contained clean sawdust and had food and water available. Weight of animals was recorded before and after the test. Food intake (FI) also was measured.

#### Days 16–21. Morris Water Maze (MWM) Test

Animals were tested for spatial learning and memory in the MWM test consisting of 1 day of cue learning and 4 days of place learning for spatial reference memory, followed by one probe trial. Mice were trained to locate a hidden platform (7 cm diameter, 1 cm below the water surface) in a circular pool for mice (Intex Recreation Corp., Long Beach, CA, United States; 91 cm diameter, 40 cm height, 25°C opaque water), located in a completely black painted 6 m^2^ test room. Mice failing to find the platform were placed on it for 10 s, the same period as the successful animals. The protocol (Giménez-Llort et al., 2007) was used as follows: 1 day of cue learning, 4 days of place learning followed by a probe trial.

##### Cue learning with a visible platform

On the first day, the animals were tested for the cue learning of a visual platform consisting of four trials in 1 day. In each trial, the mouse was gently released (facing the wall) from one randomly selected starting point (E or W) and allowed to swim until it escaped onto the platform, elevated 1 cm above the water level in the N position and indicated by a visible striped flag (5.3 × 8.3 × 15 cm). Extra maze cues were absent in the black painted walls of the room.

##### Place learning with a hidden platform

On the following day, the place learning task consisted of four trial sessions per day for 4 days with trials spaced 30 min apart. The mouse was gently released (facing the wall) from one randomly selected starting point (E or W, as these are equidistant from the target) and allowed to swim until escaped onto the hidden platform which was now located in the middle of the S quadrant. Mice that failed to find the platform within 60 s were placed on it for 10 s, the same period as was allowed for the successful animals. White geometric figures, one hung on each wall of the room, were used as external visual clues.

##### Probe trial

One hour thirty minutes after the last trial of the place learning task, the platform was removed from the maze and the mice performed a “probe trial” of 60 s to evaluate their spatial memory for the platform position.

##### Quantitative and qualitative analyses

Behavior was evaluated by both direct observation and analysis of videotape-recorded images. Variables of time (escape latency, quadrant preference), distance covered, and swimming speed were analyzed in all the trials of the tasks. The escape latency was readily measured with a stopwatch by an observer unaware of the animal’s genotype and confirmed during the subsequent video-tracking analysis. A video camera placed above the water maze recorded the animal’s behavior and thereafter an automated system (Smart, Panlab S.L., Barcelona, Spain) enabled computerized measurement of the distance traveled by the animal during the trials. The swimming speed (cm/s) of the mice during each trial was calculated. In the probe trial, the time spent in each of the four quadrants, the distance traveled along them, and the number of crossings over the removed platform position (annulus crossings) were also measured retrospectively by means of the automated video-tracking analysis.

Finally, the swim paths for each mouse in each trial of the cue learning task, place learning task, and probe trial were analyzed following the swimming strategies described by Janus (2004) and classified according to three criteria: the objective (non-search behaviors, namely floating and circling, vs. search strategies), the direction (goal-directed vs. non-goal-directed strategies), and the variety (single vs. mixed strategies) (see Baeta-Corral and Giménez-Llort, 2015).

### Survival and Immunoendocrine Status

Mortality was recorded from 6 to 13 months of age. The effects of caffeine on the neuroimmunoendocrine status (Giménez-Llort et al., 2014) were monitored by means of the levels of corticosterone and the size (weight in milligram) and relative size (% vs. BW) of the spleen (Giménez-Llort et al., 2008). Splenomegaly was used as a physical indicator of the altered status of the peripheral immune system in 3xTg-AD mice (Giménez-Llort et al., 2012; Marchese et al., 2014).

Mice were sacrificed and samples of about 0.5 ml of whole trunk blood were collected into heparinized tubes and centrifugated immediately at 10,000 × *g* for 2 min. The plasma obtained was stored at -20°C. Corticosterone content (nanogram per milliliter) was analyzed using a commercial kit (Corticosterone EIA Immunodiagnostic Systems Ltd., Boldon, United Kingdom) and ELISA EMS Reader MF V.29.-0.

### Statistics

Statistical analyses were performed using SPSS 17.0 software. All data are presented as mean ± SEM or percentage. To evaluate the effects of genotype and caffeine treatment a 2 × 2 factorial analysis design was applied. Differences were studied through Multivariate General Lineal model analysis, followed by *post hoc* Duncan’s test comparisons. *P* < 0.05 was taken as statistically significant.

## Results

**FIGURE 1:**
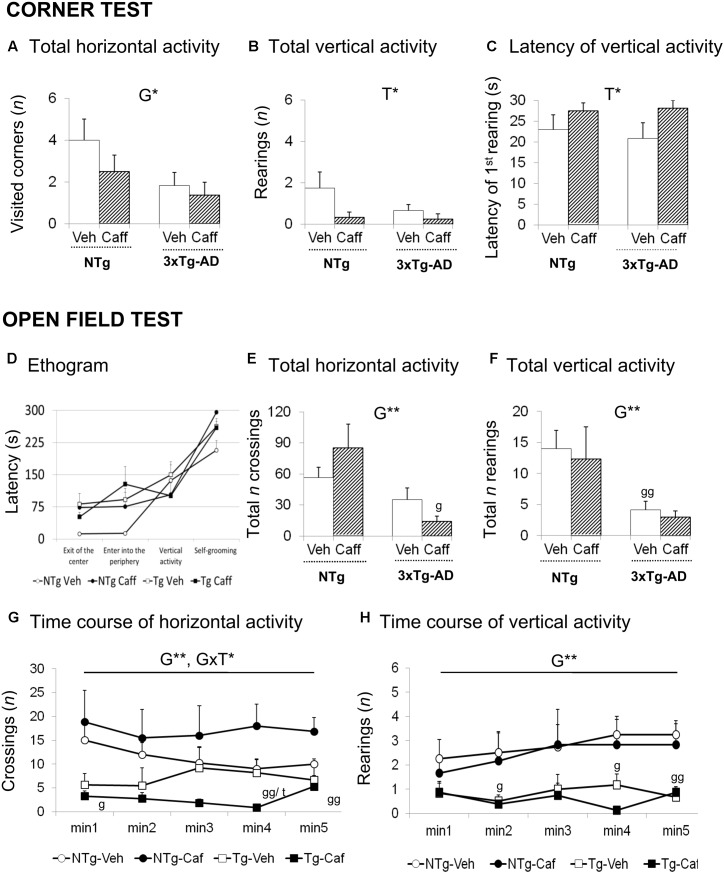
Effects of chronic caffeine treatment assessed in NTg and 3xTg-AD mice at 13-months of age in the corner (CT) and open-field (OF) tests. Horizontal **(A)** and vertical **(B,C)** activities in the corner test. Ethogram **(D)**, horizontal **(E,G)**, and vertical **(F,H)** activities in the OF test. Data are expressed by mean ± SEM. Veh, vehicle; Caff, caffeine. The text at the top of each graph refers to the *p*-values of the 2 × 2 ANOVA: G, genotype effect; T, treatment effect; GxT, genotype × treatment interaction; ^∗^*p* < 0.05, ^∗∗^*p* < 0.01, ^∗∗∗^*p* < 0.001. *Post hoc* comparisons are shown in the graphs as: g *p* < 0.05, gg *p* < 0.01 vs. the corresponding NTg group; ^∗^*p* < 0.05, ^∗∗^*p* < 0.01, ^∗∗∗^*p* < 0.001 vs. the corresponding non-treated group.

**FIGURE 2:**
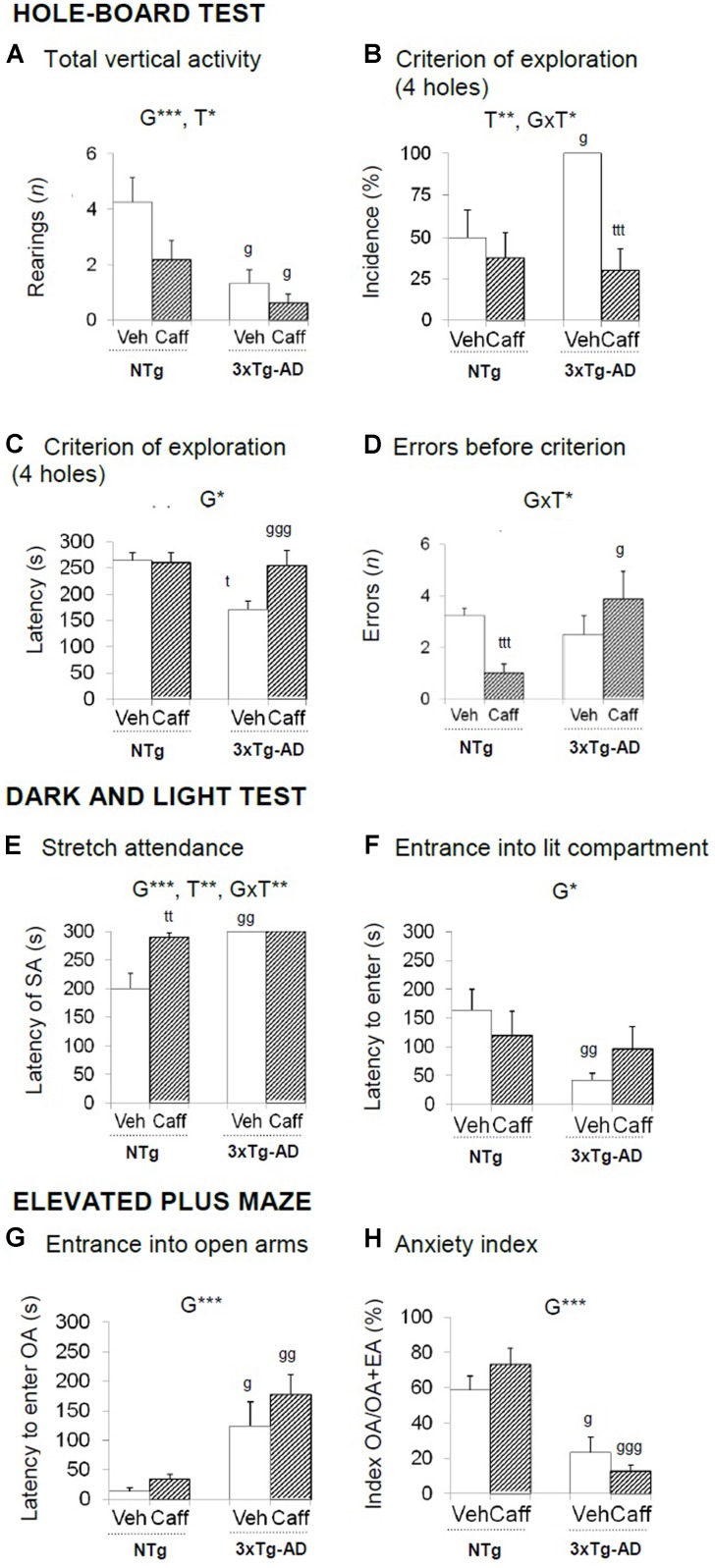
Effects of chronic caffeine treatment assessed in the HB, DLB, and EPM tests in NTg and 3xTg-AD mice at 13 months of age. Data are expressed as mean ± SEM. **(A–H)** Behavioral variables (as indicated) of the hole-board test **(A–D)**, the dark and light test **(E,F)** and the elevated plus maze **(D–H)**. The text at the top of each graph refers to the *p*-values of the 2 × 2 ANOVA: G, genotype effect; T, treatment effect; GxT, genotype × treatment interaction; ^∗^*p* < 0.05, ^∗∗^*p* < 0.01, ^∗∗∗^*p* < 0.001. *Post hoc* comparisons are shown in the graphs as: g *p* < 0.05, gg *p* < 0.01 vs. the corresponding NTg group; ^∗^*p* < 0.05, ^∗∗^*p* < 0.01, ^∗∗∗^*p* < 0.001 vs. the corresponding non-treated group. SA, stretch attendance.

**FIGURE 3:**
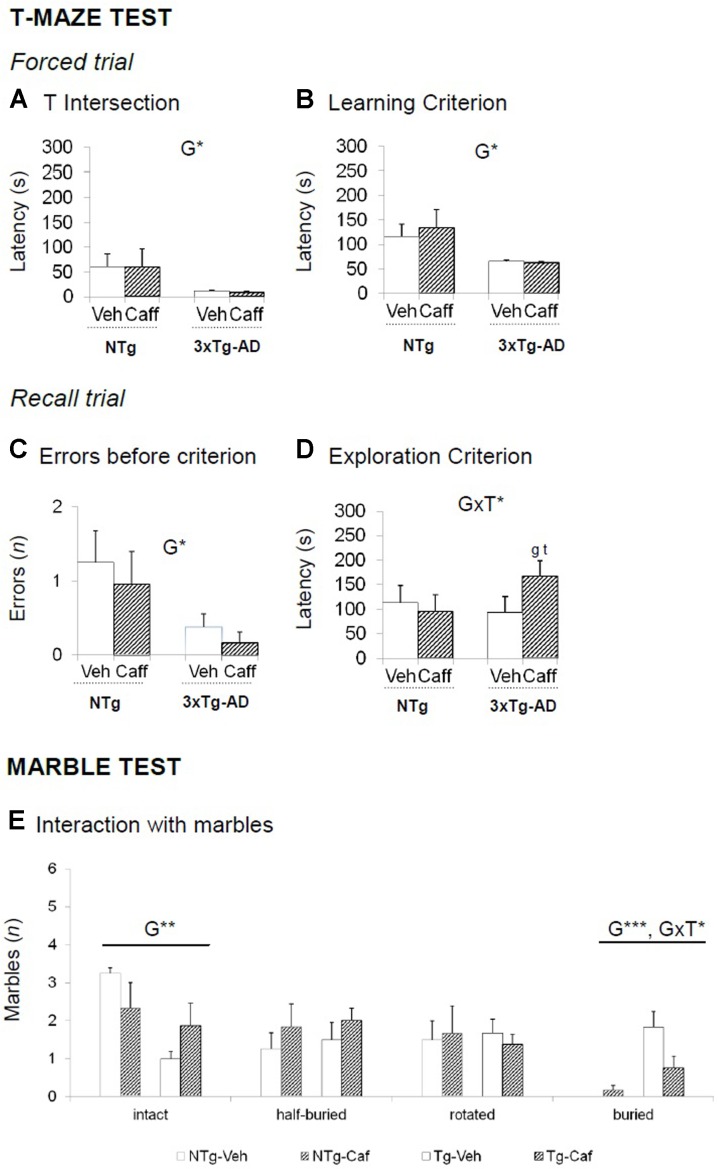
Effects of chronic caffeine treatment assessed in NTg and 3xTg-AD mice at 13 months of age in the T-maze (TM) and marble (MB) tests. **(A–E)** Behavioral variables (as indicated) of the T-maze test **(A–D)** and the marble test **(E)**. Data are expressed by mean ± SEM. Veh, vehicle; Caff, caffeine. The text at the top of each graph refers to the *p*-values of the 2 × 2 ANOVA: G, genotype effect; T, treatment effect: GxT, genotype × treatment interaction; ^∗^*p* < 0.05, ^∗∗^*p* < 0.01, ^∗∗∗^*p* < 0.001. *Post hoc* comparisons are shown in the graphs as: g *p* < 0.05, gg *p* < 0.0l vs. the corresponding NTg group: ^∗^*p* < 0.059, ^∗∗^*p* < 0.01, ^∗∗∗^*p* < 0.001 vs. the corresponding non-treated group.

**FIGURE 4:**
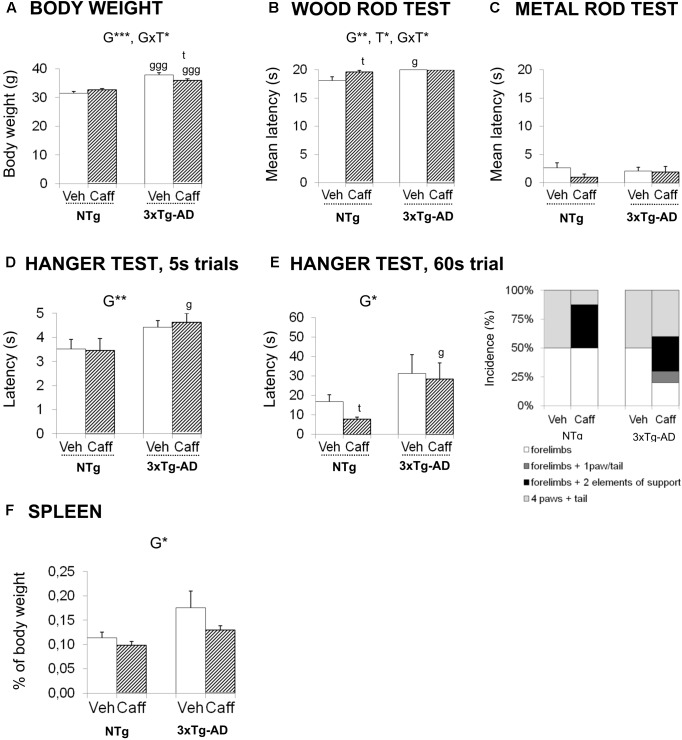
Effects of chronic caffeine treatment on body weight, sensorimotor functions, percentage of weight of spleen, and plasmatic corticosterone levels in NTg and 3xTg-AD mice at 13 months of age. **(A)** Body weight and **(B–G)** behavioral variables (as indicated) on the wood rod test **(B)**, metal rod test **(C)** and the two hanger tests **(D,E)**. The relative weight of spleen **(F)** and corticosterone levels **(G)**. Data are expressed by mean ± SEM or percentage (%). Veh, vehicle; Caff, caffeine. The text at the top of each graph refers to the *p*-values of the 2 × 2 ANOVA: G, genotype effect; T, treatment effect; GxT, genotype × treatment interaction; ^∗^*p* < 0.05, ^∗∗^*p* < 0.01, ^∗∗∗^*p* < 0.001. *Post hoc* comparisons are shown in the graphs as: g *p* < 0.05, gg *p* < 0.01 vs. the corresponding NTg group; ^∗^*p* < 0.05, ^∗∗^*p* < 0.01, ^∗∗∗^*p* < 0.001 vs. the corresponding non-treated group.

**FIGURE 5:**
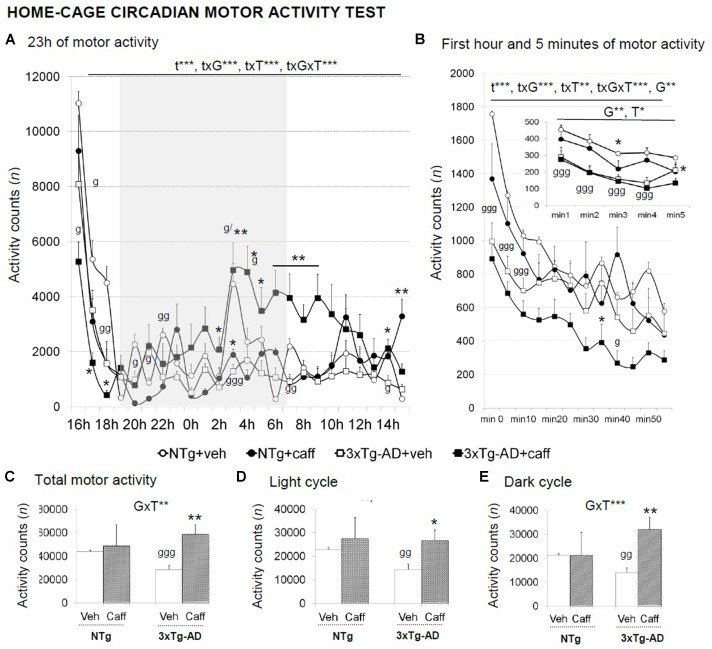
Effects of chronic caffeine treatment in the home-cage on circadian motor activity in NTg and 3xTg-AD mice at 13 months of age. Data are expressed by mean ± SEM or percentage (%). Veh, vehicle: Caff, caffeine. The vertical axis shows motor activity counts during the time intervals of the 23 h continuous recording **(A)**, 1-min intervals (right-hand graphs) during a period of l h **(B)**, or 5-min intervals (inset of **B**). Total activity counts during the 23 h **(C)**, the light **(D)**, and the dark **(E)** cycles are detailed. Data are expressed by mean ± SEM. The text at the top of each graph refers to the *p*-values of the 2 × 2 ANOVA: G, genotype effect; GxT, genotype × treatment interaction; t, time effect; txG, time × genotype interaction; txT, time × treatment interaction; ^∗^*p* < 0.05, ^∗∗^*p* < 0.01, ^∗∗∗^*p* < 0.001. *Post hoc* comparisons are shown in the graphs as: g *p* < 0.05, gg *p* < 0.01, ggg *p* < 0.00l vs. the corresponding NTg group; ^∗^*p* < 0.05 vs. the corresponding non-treated group.

**FIGURE 6:**
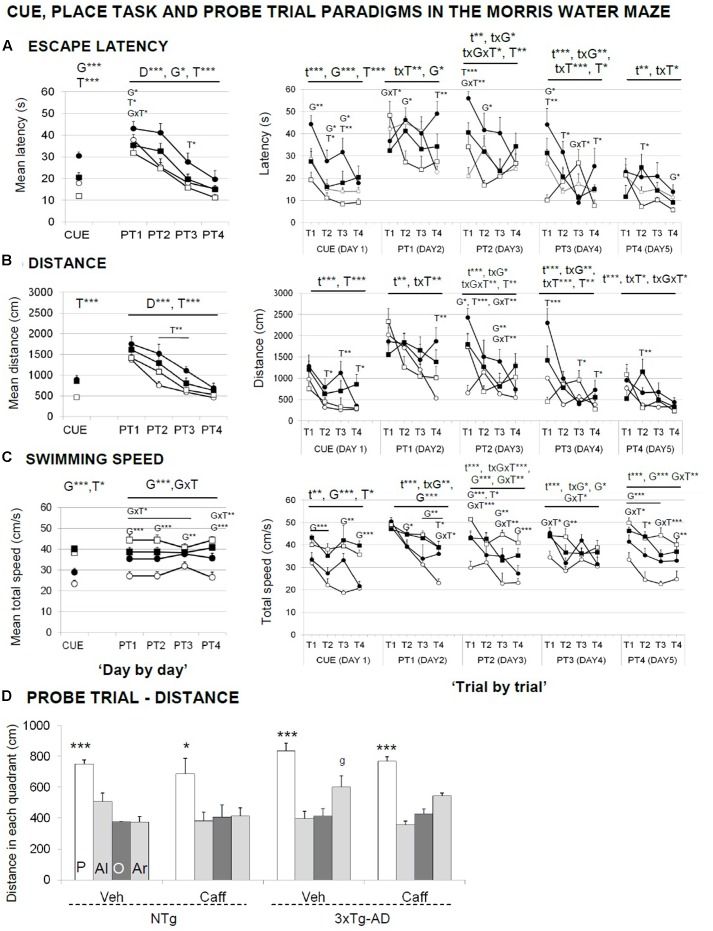
Effects of chronic caffeine treatment assessed in the CUE and PT, place learning tasks, and the probe trial of the Morris water maze test in NTg and 3xTg-AD mice at 13 months of age. Data are expressed by mean ± SEM in the cue and learning tasks **(A–C)** and by distance covered in the platform (P), adjacent left (Al), opposite (O), and adjacent right (Ar) quadrants **(D)**. ANOVA 2 × 2: G, genotype effect; T, treatment effect; GxT, genotype × treatment interaction; ^∗^*p* < 0.05, ^∗∗^*p* < 0.01, ^∗∗∗^*p* < 0.001. *Post hoc*: g *p* < 0.05 vs. the NTg group. ANOVA, ^∗^*p* < 0.05, ^∗∗∗^*p* < 0.001 P vs. all quadrants.

**FIGURE 7:**
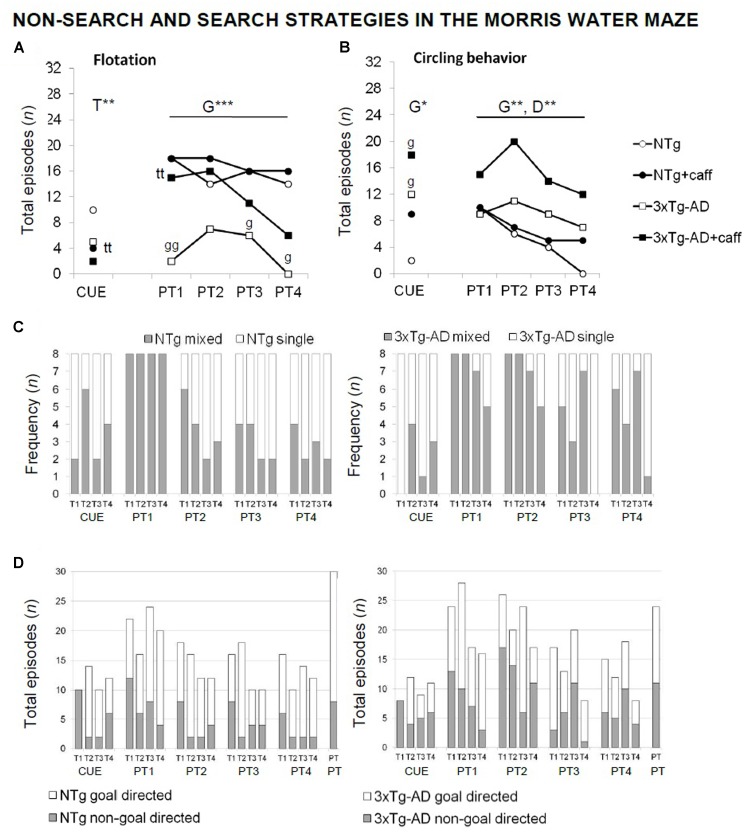
Qualitative analysis of the non-search and search strategies assessed “trial by trial” in the paradigms of the MWM test in NTg and 3xTg-AD mice at 13 months of age. Flotation behavior **(A)**, circling **(B)**, single vs. mixed strategies **(C)**, and goal- vs. non-goal-directed strategies **(D)**. Data are expressed by frequency or total number of episodes (*n*). RMA: G, genotype effect; D, day effect; ^∗^*p* < 0.05, ^∗∗^*p* < 0.01. *Post hoc*: ^∗^*p* < 0.05, ^∗∗^*p* < 0.0l vs. the corresponding NTg group.

**Figures 1**–**7** summarize the behavioral phenotype exhibited by male 3xTg-AD and NTg at 13 months of age and the effects of caffeine on these behaviors.

### Corner Test (CT)

Genotype and treatment effects were found in the CT (**Figures 1A–C**). Horizontal locomotor activity measured by number of corners visited was reduced in the 3xTg-AD mice [G, *F*(1,30) = 4.760; *p* < 0.05] as compared to the NTg animals. Vertical activity was influenced by caffeine, with treated animals showing higher latencies to perform a first rearing [T, *F*(1,30) = 4.676; *p* < 0.05] and a reduction in the total number of rearings [T, *F*(1,30) = 4.571; *p* < 0.05].

### Open-Field Test

Genotype differences were found in the ethogram (**Figure 1D**), the behavioral sequence of events, pointing out to increased thigmotaxis. 3xTg-AD animals spent more time leaving the center of the apparatus [G, *t*(1,14) = -2.785; *p* < 0.05] and arriving at the periphery [G, *F*(1,30) = 4.366; *p* < 0.05]. Caffeine increased the time spent in the center in NTg+caff animals whereas it was reduced in the 3xTg-AD group [GxT, *F*(1,30) = 4.936; *p* < 0.05]. Once the animals arrived to the periphery, self-grooming behavior was delayed in time in NTg+caff mice as compared to their control group [T, *F*(1,30) = 7.158; *p* < 0.05 and GxT, *F*(1,30) = 8.194; *p* < 0.01] although the total duration of self-grooming (NTg-Veh: 2.25 s ± 0.48; NTg-Caff: 1.00 s ± 0.50; Tg-Veh: 1.50 s ± 0.50; Tg-Caff: 1.75 s ± 0.52) was not modified.

Regarding the locomotor activity (**Figures 1E–H**), 3xTg-AD mice showed a reduced number of crossings as compared to NTg animals [G, *F*(1,30) = 12.132; *p* < 0.01]. Moreover, caffeine had a bidirectional effect increasing this horizontal component in the NTg genotype whereas reducing it in the 3xTg-AD treated animals [minute 4; GxT, *F*(1,30) = 8.994; *p* < 0.01]. In the vertical activity, 3xTg-AD mice showed a reduced number of rearings as compared to the NTg mice [G, *F*(1,30) = 10.944 *p* < 0.01]. No stereotyped rearing was observed in NTg mice (NTg-Veh: none) and their presence was scarce in the other groups (NTg-Caff: 0.33 ± 0.30; Tg-Veh: 0.17 ± 0.10; Tg-Caff: 1.25 s ± 0.90).

Finally, the NTg+caff group showed an increase in defecation behavior whereas it was reduced in the 3xTg-AD+caff group [(NTg-Veh: 2.00 s ± 0.20; NTg-Caff: 3.50 ± 0.20; Tg-Veh: 3.67 ± 1.0; Tg-Caff: 2.50 ± 0.62) GxT, *F*(1,30) = 4.681; *p* < 0.05].

### Hole-Board Test

Significant changes in the exploratory activity were detected in the HB test (**Figures 2A–D**) All groups showed similar latencies in the first movement and to explore the first hole. The reduction in the exploratory activity in the 3xTg-AD groups was the most significant difference observed in this test [G, *F*(1,30) = 13.492; *p* < 0.001]. Treatment reduced vertical activity in both genotypes [T, *F*(1,30) = 5.290; *p* < 0.05]. The 3xTg-AD groups performed more head dippings [G, *F*(1,30) = 4.750; *p* < 0.05] and total time spent in this activity was higher as compared to the NTg groups [G, *F*(1.30) = 4.818; *p* < 0.05]. Hundred percent of the 3xTg-AD groups reached the criterion of the four holes exploration [G, *t*(1,14) = -3.055; *p* < 0.05] and faster than NTg groups [G, *F*(1,30) = 4.893 *p* < 0.05]. Independently of the genotype, caffeine reduced the percentage of animals that reached the criterion [T, *F*(1,30) = 11.904; *p* < 0.01]. Moreover, the treatment increased the number of errors in the 3xTg-AD genotype whereas it was reduced in the NTg+caff animals [GxT, *F*(1,30) = 5.652; *p* < 0.05].

Grooming behavior was advanced in time in 3xTg-AD the spent more time on it [G, *F*(1,30) = 7.649; *p* < 0.01 and G, *F*(1,30) = 7.179; *p* < 0.05, respectively]. Finally, caffeine reduced the number of defecation boli [T, *F*(1,30) = 5.457; *p* < 0.05] especially in the 3xTg-AD mice [GxT, *F*(1,30) = 6.365; *p* < 0.05].

### Dark–Light Box Test

Stretch attendance activity (**Figures 2E,F**) was present in NTg but not in 3xTg-AD mice [G, *F*(1,30) = 17.690; *p* < 0.001] and caffeine increased the latency of stretch attendance in the NTg mice [T, *F*(1,30) = 11.842; *p* < 0.01 and GxT, *F*(1,30) = 11.842; *p* < 0.01].

The incidence of animals that entered into the lit area ranged 50–70% in the NTg mice and increased to the 90–100% in the 3xTg-AD mice [G, *F*(1,30) = 9.098; *p* < 0.01]. The disinhibitory behavior of the 3xTg-AD groups was shown as a reduced latency to enter into the lit area [**Figure 4B**; G, *F*(1,30) = 4.859; *p* < 0.05], more than double of crossings between the two compartments [G, *t*(1,14) = -3.049; *p* < 0.01], and less time into the lit area [G, *F*(1,30) = 4.158; *p* < 0.05].

Finally, a genotype and a genotype × treatment interaction effect was found in total defecation [G, *F*(1,30) = 4.158; *p* < 0.05 and GxT, *F*(1,30) = 6.794; *p* < 0.05, respectively].

### Elevated Plus Maze

The latency to enter into the OA [G, *F*(1,30) = 20.029; *p* < 0.001] and the anxiety index TOA/(TOA+TEA) [G, *F*(1,30) = 43.619; *p* < 0.001] indicated that 3xTg-AD animals were more anxious than NTg mice (**Figures 2G,H**). All groups showed a similar number of entries in all the arms and the central piece [all *F*s(1,30) < 3.583; *p* > 0.068, n.s.]. A genotype and a genotype × treatment interaction effects were found in defecation behavior [G, *F*(1,30) = 4.536; *p* < 0.05 and GxT, *F*(1,30) = 6.648; *p* < 0.05, respectively].

### T-Maze Test

In the forced trial, 3xTg-AD groups spent less time to reach the intersection point of the TM [G, *F*(1,30) = 5.729; *p* < 0.05] and to reach the criterion of the 20 s exploration [G, *F*(1,30) = 56.375; *p* < 0.05]. In the recall, the number of errors before choosing the unexplored arm in the previous trial was lower in the 3xTg-AD genotype [G, *F*(1,30) = 6.111; *p* < 0.05]. However, 3xTg-AD-treated animals reduced their efficiency to explore both arms since they spent more time to reach the goal [GxT, *F*(1,30) = 4.188; *p* < 0.05] (**Figures 3A–D**).

### Marble Test

The 3xTg-AD mice buried a higher number of marbles [G, *F*(1,28) = 20.802; *p* < 0.001] whereas NTg animals left them intact [G, *F*(1,28) = 8.660; *p* < 0.01]. Caffeine reduced the number of marbles buried in the 3xTg-AD genotype [GxT, *F*(1,28) = 5.565; *p* < 0.05] (**Figure 3E**).

### Body Weight and Sensorimotor Functions

At 6 months of age, before the treatment was started, the 3xTg-AD mice were overweighed (+20.26%) [t, *F*(1,32) = -5.603, *p* < 0.000]. The genotype effect was maintained till the end of the treatment (+20.09%) [G, *F*(1,30) = 68.826; *p* < 0.001].

At 13 months of age, the genotype × treatment interaction effects [GxT, *F*(1,30) = 7.383; *p* < 0.05] pointed out a reduction of the BW induced by caffeine in the 3xTg-AD animals (Duncan’s test, *p <* 0.05 vs. 3xTg-AD+veh group) but the treatment did not correct the overweight of 3xTg-AD mice (Duncan’s test, *p* < 0.05 vs. NTg+caff).

In the sensory-motor functions, no deficits were found in the reflexes assessed, with all the animals obtaining the maximum score. In the wood rod test, most animals petrified (no distance covered) and this response determined a high latency to fall. Still, 3xTg-AD mice exhibited longer latencies to fall than NTg animals [G, *F*(1,30) = 12.037; *p* < 0.01]. Caffeine increased the latency to fall in the NTg genotype [T, *F*(1,30) = 4.841; *p* < 0.05]. When the complexity of the task was increased (metal wire test) all groups showed worse equilibrium, but no differences were found between the groups neither in the latency to fall nor in the distance covered. In the Hanger test, the 5 s trial showed genotype-dependent differences in the latency to fall [G, *F*(1,30) = 7.879; *p* < 0.01]. This effect was confirmed in the 60 s trial [G, *F*(1,30) = 6.561; *p* < 0.05] (**Figures 4A–E**).

### Circadian Motor Activity Test

A circadian temporal course was found in the 23 h motor activity period studied [t, *F*(23,690) = 29.732; *p* < 0.001] that differed between genotype [txG, *F*(23,690) = 4.570; *p* < 0.001], treatment [txT, *F*(23,690) = 5.360; *p* < 0.001], and the interaction between these two factors [txGxT, *F*(23,690) = 2.858; *p* < 0.001] (**Figure 5A**). During the first hour of habituation, time [t, *F*(11,330) = 78.341; *p* < 0.001] and genotype [G, *F*(11,330) = 9.395; *p* < 0.01] effects were found. Besides, “time × genotype” [txG, *F*(11,330) = 7.984; *p* < 0.001], “time × treatment” [txT, *F*(11,330) = 2.591; *p* < 0.01], and “time × genotype × treatment” [txGxT, *F*(11,330) = 4.083; *p* < 0.001] interaction effects were found (**Figure 5B**) with treated 3xTg-AD mice showing a reduced locomotor activity as compared to their non-treated group.

3xTg-AD+veh mice showed a reduced total motor activity [G, *t*(1,15) = 6.591; *p* < 0.01]. Caffeine increased the overall spontaneous motor activity along a 23-h LD period in the 3xTg-AD genotype [**Figure 5C**; GxT, *F*(1,30) = 11.525; *p* < 0.01], and more significantly, during the dark cycle [**Figure 5E**; *F*(1,30) = 15.311; *p* < 0.001].

### Morris Water Maze Test

**Figures 6A–C** illustrate the “day-by-day” (left panel) and “trial-by-trial” (right panels) acquisition curves.

In the cue learning task (**Figure 6A**, CUE), genotype and treatment effects were found, with 3xTg-AD mice reaching the visible platform faster than NTg [G, *F*(1,30) = 17.727; *p* < 0.001]. Independently of the genotype, treated animals spent more time [T, *F*(1,30) = 30.891; *p* < 0.001] and showed longer distance covered [T, *F*(1,30) = 28.171; *p* < 0.001] to find the platform than non-treated mice. 3xTg-AD mice showed an increased swimming speed [G, *F*(1,30) = 68.397; *p* < 0.001] and caffeine increased the swimming speed in both treated groups [T, *F*(1,30) = 5.394; *p* < 0.05].

In the place learning task (PT), when the cue was removed and the platform was hidden, animals exhibited a different genotype- and treatment-dependent acquisition curves, with 3xTg-AD animals finding faster the hidden platform along the 4 days of the test [G, *F*(1,30) = 6.920; *p* < 0.05]. Caffeine increased the time spent [T, *F*(1,30) = 11.449; *p* < 0.01] and the distance covered [T, *F*(1,30) = 15.566; *p* < 0.001] to reach the platform as compared to their non-treated groups. Swimming speed showed a consistent genotype effect [G, *F*(1,30) = 21.239; *p* < 0.001]. Caffeine modified the swimming speed in an opposite manner, since it was increased in the NTg and reduced in the 3xTg-AD animals [GxT, *F*(1,30) = 9.540; *p* < 0.05].

“Trial-by-trial” analysis revealed that time, genotype, and treatment factors frequently showed mutual interactions [**Figures 6A–C**, right panels; RMA, *F*(3,90) > 2.984; *p* < 0.05]. Between all the differences found, it is interesting to note that caffeine effects were found both in long-term (T1) and short-term (T3 and T4) memory trials. The acquisition level achieved at the end of the place task (distance PT4 and PT4.4) was similar in all the groups [G and T, *F*(1,30) < 1.631; *p* > 0.05, n.s.].

In the probe trial (**Figure 6D**), all the groups showed similar ability to distinguish the platform quadrant during the place task [all ANOVAs, *F*(3,28) > 25.522; *p* < 0.001] despite the NTg+caff group did it with one or two lower scale [all ANOVAs, *F*(3,28) > 3.667; *p* < 0.05].

Qualitative analysis of the non-search behaviors and search strategies allowed to find caffeine effects (**Figure 7**) based on the distinctive characteristics of both genotypes: presence of floating and the use of “single-” and “goal-directed” strategies in the NTg genotype in contrast to “circling” and “mixed” and “non-goal-directed” strategies in the 3xTg-AD mice. Caffeine modified these swimming patterns, reducing differences between genotypes. Thus, the NTg+caff group showed a higher proportion of “mixed” and “non-goal-directed” strategies whereas the 3xTg-AD group showed more “single-” and “goal-directed” strategies.

Moreover, in the cue learning task, caffeine reduced the incidence of floating [T, *F*(1,30) = 7.660; *p* < 0.01] and increased the incidence of “thigmotaxis” (test exacte de Fisher; *p* < 0.05) in both treated groups.

In the probe trial, all the vehicle animals (100%) swam “directly” to the platform quadrant during the place task, whereas nearly 50% of the 3xTg-AD+caff animals used “random search.” When the animals failed to find the platform, NTg+veh mice alternated different strategies. In contrast, 3xTg-AD mice persisted in their behavior. A higher variety of strategies was shown by both genotypes of treated animals.

### Survival and Immunoendocrine Status

All NTg+veh mice survived until the age of 13 months, whereas the survival rate in the 3xTg-AD+veh and NTg+caff groups of the same age decreased to 80%. However, the differences were not significant when analyzed statistically.

The weight of the spleen was increased in 3xTg-AD mice [G, *F*(1,30) = 6.549; *p* < 0.05]. In the treated groups, caffeine showed a tendency to reduce the weight of the spleen [T, *F*(1,30) = 2.721; *p* = 0.109, n.s.]. In 3xTg-AD+caff mice, this reduction was sufficient to restore the normal weight of the spleen [G, *t*(1,16) = -1.145; *p* = 0.269, n.s. vs. the control group NTg+veh] (**Figure 4F**).

Corticosterone levels showed slight increases due to genotype [G, *F*(1,30) = 1.983; *p* = 0.169, n.s.] that did not reach statistical significance. If any, treatment [T, *F*(1,30) = 0.728; *p* = 0.400, n.s., GxT, *F*(1,30) = 0.311, *p* = 0.400, n.s.] slightly trend to increase corticosterone levels in 3xTg-AD+caff mice.

## Discussion

This study analyzes, in a translational scenario, the long-term effects of a chronic low dose of caffeine started at the onset of disease (6 months of age) in 3xTg-AD mice, an animal model for Alzheimer’s disease characterized by cognitive but also BPSD-like profile (Giménez-Llort et al., 2006). The behavioral effects were assessed at advanced stages (13 month of age) when both amyloid and tau pathologies are present (Oddo et al., 2003). At this age, we have consistently reported that survival male 3xTg-AD mice starts to be compromised (Giménez-Llort et al., 2008, 2010; García-Mesa et al., 2016; Torres-Lista et al., 2017). Effects were compared to age-matched NTg mice with normal aging, that according to the background strain represent overcoming the middle age. The results showed significant effects of caffeine in most of the variables, especially those related to neophobia and other anxiety-like behaviors, emotionality, and cognitive flexibility. Thus, anxiogenic effects were seen in middle-aged animals and that effect, in the 3xTg-AD model, resulted in an aggravation of its BPSD-like pattern. The groups treated with caffeine did not improve their long-term memory until they completed the behavioral spatial reference memory paradigm in the water maze, and the short-term memory, in any case, was disadvantaged. It was only in the second time interval of the probe trial, where the 3xTg-AD group treated with caffeine was able to use search strategies similar to those exhibited by both groups of NTg mice. In addition, the behavioral analysis pointed at distinct genotype-dependent functional capacity of caffeine-treated animals to meet task-dependent performance demands. Thus, selective effects of caffeine for the 3xTg-AD genotype were observed in the increase of the circadian motor activity and the reduction of body and spleen weights, indicators of the functional and neuroimmune status. Caffeine also exerted bidirectional effects: stimulating motor activity in NTg mice in the open-field (OF) test but reducing it in the 3xTg-AD; increasing the emotionality of NTg mice and decreasing it in the 3xTg-AD in the OF, EPM, and HB; and finally, modifying the navigation strategies in the learning tasks of the MWM, making them more similar.

The anxiogenic effects induced by caffeine were observed, in general, as an increase of neophobia and the anxious profile. In the NTg genotype, the reduction in the exploratory behavior in CT, the increased latency to reach the protected areas (thigmotaxis) in the OF, and the delay in the risk assessment activity in the DLB demonstrated these anxiogenic effects. The increase in defecation observed in the NTg+caff group suggests an increased emotionality induced by caffeine. These results agree with those obtained in animals treated with high doses of caffeine, which were more emotionally reactive and showed more immobility, defecation, and urination than control animals (Anderson and Hughes, 2008). In 3xTg-AD animals, increased anxiety profile induced by chronic caffeine treatment led to a worsening BPSD pattern, where the behavioral response varied depending on the level of anxiety that each test involves. In this regard, direct exposure to an open and illuminated field caused a reduction in motor activity, almost completely. Conversely, in mild stressful environments such as the case of the motor ACT, the anxious response was reflected as an increase in the hyperactive pattern characteristic of 3xTg-AD animals (Giménez-Llort et al., 2007). In the cue learning task of the MWM, this increase of the hyperactivity pattern induced by caffeine was also observed as an increase in the swimming speed as compared to the 3xTg-AD+veh group, that may explain the reduction of floating behavior. The stimulating effects of caffeine at the motor level were observed in NTg animals as an increase in the number of crossings in the OF and the swimming speed in the learning tasks of the MWM. As expected (i.e., Nehlig et al., 1992), this stimulatory effect of the horizontal motor activity was in decrement to the vertical activity, considered the variable that better reflects the exploratory behavior *per se* (Colorado et al., 2006). Therefore, caffeine exerted its effects increasing hyperactivity (locomotion) and reducing vertical exploratory behavior.

As introduced before, here it is interesting to note that a depressive-like profile paired to monoaminergic alterations has been recently reported in the 3xTg-AD mice using two models of stress-coping behavior (FST, Porsolt forced swim test, and Tail suspension test) and with an anhedonia test such as the sucrose preference test (Romano et al., 2015). In the current study, the effects of caffeine on behavioral despair were not directly addressed, i.e., using the forced swim test in order to avoid carry on effects on the MWM. Also, because in our hands 3xTg-AD mice showed a persistence of behaviors in the FST that interfered with the interpretation of the performances (Torres-Lista and Giménez-Llort, 2014, 2015). Instead, the presence of immobility (floating) was taken into account in all the trials in the maze (as described in Baeta-Corral and Giménez-Llort, 2015) and the “Cue learning with a visible platform” was a specific paradigm used to control lack of motivation as well as sensorimotor differences. Besides, the effect of caffeine on other variables such as exploration in the ACTs and more specifically the performance in the HB test for novelty seeking was among the studied behaviors as opposed to the expression of apathy/depressive symptoms.

Regarding sensorimotor functions, the results obtained in the balance of 3xTg-AD mice cannot exclude the presence of a false positive, since the innate fear of heights made that group showing more petrifaction (i.e., genotype 3xTg-AD treated with caffeine) were those that stayed longer on the rod. This is in agreement with prior results obtained at the same age, in female 3xTg-AD mice (Giménez-Llort et al., 2007). In contrast, in NTg animals that roam the rod, caffeine improved balance but worsened muscle resistance in this genotype. Similar results were also obtained by our laboratory in the behavioral assessment A_1_ receptor knockout mice (Giménez-Llort et al., 2002), a genetic strategy to emulate the chronic effects of caffeine (Johansson et al., 2001).

It has been shown that chronic caffeine treatment prevents weight gain in rodents that were fed a high fat diet (Moy and McNay, 2013). In the present work, the long-term treatment with a low dose of caffeine modified, but not corrected, the overweight of 3xTg-AD mice. We have already shown that overweight is a characteristic of the Spanish colony of 3xTg-AD mice, since onset of disease (Giménez-Llort et al., 2010), it is related to a higher relative contribution of white adipose tissue (WAT) (Giménez-Llort et al., 2010; García-Mesa et al., 2011, 2012) and could not be corrected by health strategies such as forced (Giménez-Llort et al., 2010) or voluntary exercise (García-Mesa et al., 2011). In the present work, the increase in the nocturnal activity found in 3xTg-AD+caff mice could explain their weight loss.

The MWM showed that the increased latency, distance, and speed that chronic caffeine indiscriminately exerts over both genotypes in the cue learning task does not correspond to the expected cognitive effects, quite the contrary. In the first experience in the maze, the benefits attributed to caffeine improving attention (Griffiths et al., 1990), did not confer any advantage to the animals in this learning task, considered as a visual perceptive learning. In the following three trials for short-term memory, the effects were also contrary to those expected, since caffeine increased the distance covered to reach the platform. In the second paradigm, the place learning task, increased speed in the NTg group and decreased in the 3xTg-AD could emulate stimulant and depressant effects of low and high doses of caffeine, respectively (Fredholm et al., 1999). Thus, the chronic low-dose (0.3 mg/kg) acted exhilarating swimming speed in the NTg group, while in 3xTg-AD mice – which consistently show a higher speed than NTg animals – the reduction induced by caffeine may be the result of a depressant drug effect. Although it seems that caffeine improved short-term memory because it did so in a pair of trials, this fact could be considered exceptional in the face of nine trials in which the effects of caffeine involved a significantly worse execution. At the end of this task, all experimental groups reached the same level of acquisition and in the probe trial, conducted after 1 h 30 min, all of them also showed the same ability to remember the position of the final platform. Still, the NTg+caff group did so with one or two lower orders of magnitude. Considering that in the first trials of everyday, quantitative values between NTg and 3xTg-AD were more distinct, it is likely that a 24-h probe trial would have been more suitable to detect cognitive differences. In general, quantitative results show that, under these experimental conditions, cognitive outcomes were strongly conditioned by the genotype differences in swimming speed or the hyperactive profile shown in our 3xTg-AD colony.

The anxiogenic conditions that the MWM represents for mice were also reflected in the high level of floating observed in NTg animals and the sustained increase in the speed of 3xTg-AD mice. As we have extensively discussed in a precedent report’ (Baeta-Corral and Giménez-Llort, 2015), this means that in this colony of 3xTg-AD mice, the MWM may probably not be specific to assess hippocampal-dependent cognitive deficits related to spatial memory, as in other models for AD (i.e., Arendash et al., 2009). In this mice model, the MWM may involve the assessment of cognition under anxiogenic conditions and therefore the measurement of emotional memory depending on limbic system. Therefore, the anxiogenic effects of caffeine may have counteracted the potential cognitive effects in both visual perceptive (cue learning task) and spatial (place learning task) learning and memory tasks. The fact that the acquisition curve of the 3xTg-AD animals showed an even better performance than NTg mice reminds us of the results obtained in this colony of animals in the conditioned fear test (España et al., 2010). In that work, not only the 3xTg-AD model but also APP_Swe_ and APP_Swe_/ind models showed an enhanced contextual conditioned fear response that was dependent on their respective levels of accumulation of βA in the basolateral amygdala.

The possible masking that the presence of flotation could exert on the measures of latency and distance was also considered. The analysis of these variables including the time invested in flotation indicated that results did not differ from those obtained when the total floating time was excluded. Regarding this “non-search behavior,” caffeine reduced the incidence of floating in the cue learning task in both genotypes. This action could be explained by its effects increasing attention or motivation in this learning and memory visual perceptive task. The effects of increasing the incidence of “thigmotaxis,” that is a non-goal-directed swimming around the wall of the pool, would be consistent with the horizontal locomotor hyperactivity induced by caffeine in the OF.

In order to better understand the results shown in the MWM, we analyzed the swimming strategies developed along the different trials of the three paradigms (Baeta-Corral and Giménez-Llort, 2015). The detailed analysis of strategies unveiled traits that allowed to distinguish both genotypes: single- and goal-directed strategies in NTg animals but mixed and non-goal-directed in the 3xTg-AD ones. In the present work, caffeine decreased genotype differences in learning and memory tests, because the NTg-treated animals showed mixed and non-goal-directed strategies and, conversely, the 3xTg-AD exhibited single- and goal-directed strategies attributed to a normal pattern. Therefore, behaviors that were previously easy discriminated, now were more similar.

In the probe trial, two intervals could be distinguished: the first section of navigation until the animals arrive to the previous location of the platform, and the remaining interval in which the animals could look for it or not in a new location. While in the first interval, all animals, 3xTg-AD and NTg, swam directly to the platform, caffeine treatment reduced in 50% the use of this strategy in the 3xTg-AD+caff group. In the second interval could be hypothesized that the animals are facing a problem similar to the first day of the place task, with the exception that now there have already fulfilled the acquisition process. Here, the 3xTg-AD mice showed a poor cognitive flexibility using steadily a single strategy, which could be considered an inefficient response to solve this situation. Interestingly, we have reported poor cognitive flexibility shown as persistence of behaviors in the forced swim test at more advanced stages of disease (17 months of age) (Torres-Lista and Giménez-Llort, 2014). In this sense, it is important to note that caffeine increased the variety of strategies in the 3xTg-AD group suggesting improved cognitive integration processes that may be taking part in the resolution of the problem.

Regarding mortality data in this study, the number of animals is far from the minimum necessary to reliably assess the degree of survival and the effects of caffeine on it. Still, what our results suggest is that the data are congruent with the increased vulnerability of male 3xTg-AD mice at neuroimunoendocrine level, that could explain an important 40% of mortality at 12–13 months of age (Giménez-Llort et al., 2008) that can reach 100% at 15 month of age (García-Mesa et al., 2016). The observed mortality in the NTg+caff group would be in agreement with the reduced survival curve we reported in A_1_ knockout mice (Giménez-Llort et al., 2002). While health benefits of caffeine and coffee are increasingly recognized, there are also notable reports of adverse effects of especially high-dose caffeine (Jain et al., 2017), including a case report of psychotic symptoms in a patient with dementia (Golden et al., 2015). The neurochemical scenario produced by long-term loss of A1 and A2a receptor function has been addressed in A1 (Johansson et al., 2001) and A2a (Ledent et al., 1997) knockout mice. Perhaps adverse effects can be sufficiently avoided by partial receptor blockade by low doses. Already now, there is reason to consider caffeine intake in patients with BPSD and its reduction in difficult-to-treat cases.

Since our first report (Giménez-Llort et al., 2008) proposing gender-specific immunoendocrine aging in 3xTg-AD mice, we have consistently reported that simple measures of weight and relative weight of peripheral organs indicate splenomegaly and thymus involution in this AD model. Both are considered physical indicators of peripheral immunological system aging (reviewed by Giménez-Llort et al., 2012) and impaired neuroimmunoendocrine crosstalk in AD (Giménez-Llort et al., 2014). More recently other laboratories have worked on this issue and successfully demonstrated the validity of splenomegaly as part of the autoimmune manifestations in the 3xTg-AD model (Marchese et al., 2014). In the present work, relative spleen size was slightly modulated by caffeine in both genotypes, a modulatory effect enough to restore the normal weight of the spleen in the 3xTg-AD mice. This suggests that in the 3xTg-AD+caff group, there could be an improvement in the deregulation of this network that recently has been described as relevant in AD (Giménez-Llort et al., 2014).

At the endocrine level, slight increases of corticosterone were observed due to genotype and treatment, without reaching statistical significance. This trend would be in agreement with our first report on the increase of glucocorticoid levels in male 3xTg-AD mice at more advanced stages of disease, concomitantly to increased anxiety and peripheral immune dysfunction (Giménez-Llort et al., 2008). Stress-like patterns of increased corticosterone secretion and decreased thyrotropin are described among the neuroendocrine effects of caffeine, while chronic treatment is known to induce tolerance to these effects (Spindel et al., 1983).

Immunomodulatory effects of caffeine by the decrease of cytokines (Frost-Meyer and Logomarsino, 2012) have also been proposed to contribute to neuroprotection, e.g., in Alzheimer’s disease (Horrigan et al., 2006). A better balance between pro- and anti-inflammatory cytokines in favor of anti-inflammation is also posed as the main hypothesis to explain the effects of caffeine reducing the inflammatory processes in severe life-threatening conditions (Bessler et al., 2012). Further experiments addressing the effects of chronic caffeine on peripheral cytokine levels will help to better elucidate its actions on the impaired neuro-immune system in AD.

## Conclusion

The present results provide evidence of the adverse effects of caffeine in 3xTg-AD mice with a BPSD-like profile that raises the concern for its general recommendation to AD patients. These results confirm that caffeine, despite its everyday use and relative lack of government regulation, is a potent compound with multifaceted effects. Our study adds to the evidence for caffeine and other adenosine-receptor blockers have distinct physiological effects. Some ways to deal with these multi-effects are to optimize the dose, to use active substances in coffee other than caffeine, and to use synthetic drugs modeled after caffeine, such as subtype-selective adenosine receptor antagonists, rather caffeine itself. We speculate that over a chronic treatment with caffeine, the exacerbation of anxiety-like BPSD symptoms may partially interfere with the beneficial cognitive effects to the extent that they can be in the opposite direction.

## Author Contributions

LG-L the concept development, the study design, the study conduct, and the data collection. RB-C data analysis. RB-C and LG-L data interpretation and drafting the manuscript. BJ scientific discussions and critical revision of the manuscript and figures content. All authors approved final version of the manuscript. LG-L and BJ supported for financial resources.

## Conflict of Interest Statement

The authors declare that the research was conducted in the absence of any commercial or financial relationships that could be construed as a potential conflict of interest.
